# Positive Programmed Cell Death-Ligand 1 Expression Predicts Poor Treatment Outcomes in Esophageal Squamous Cell Carcinoma Patients Receiving Neoadjuvant Chemoradiotherapy

**DOI:** 10.3390/jcm8111864

**Published:** 2019-11-03

**Authors:** Wan-Ting Huang, Hung-I Lu, Yu-Ming Wang, Yen-Hao Chen, Chien-Ming Lo, Wei-Che Lin, Ya-Chun Lan, Ling-Huei Tseng, Shau-Hsuan Li

**Affiliations:** 1 Department of Pathology, Kaohsiung Chang Gung Memorial Hospital and Chang Gung University College of Medicine, Kaohsiung 83301, Taiwan; huangwanting5@gmail.com; 2 Affiliation 2; Department of Medical Laboratory Sciences and Biotechnology, Fooyin University, Kaohsiung 83102, Taiwan; 3 Department of Thoracic and Cardiovascular Surgery, Kaohsiung Chang Gung Memorial Hospital and Chang Gung University College of Medicine, Kaohsiung 83301, Taiwan; luhungi@yahoo.com.tw (H.-I.L.); t123207424@cgmh.org.tw (C.-M.L.); 4 Department of Radiation Oncology, Kaohsiung Chang Gung Memorial Hospital and Chang Gung University College of Medicine, Kaohsiung 83301, Taiwan; scorpion@cgmh.org.tw; 5 Department of Hematology-Oncology, Kaohsiung Chang Gung Memorial Hospital and Chang Gung University College of Medicine, Kaohsiung 83301, Taiwan; alex2999@cgmh.org.tw (Y.-H.C.); amylan1226@gmail.com (Y.-C.L.); libbycatblue@gmail.com (L.-H.T.); 6 Department of Diagnostic Radiology, Kaohsiung Chang Gung Memorial Hospital and Chang Gung University College of Medicine, Kaohsiung 83301, Taiwan; u64lin@yahoo.com.tw

**Keywords:** esophageal cancer, squamous cell carcinoma, PD-L1, chemoradiotherapy, immunotherapy

## Abstract

Background: Programmed cell death-ligand 1 (PD-L1) is present in a subgroup of cancer patients who may be favorable targets for immune checkpoint inhibitor therapies. However, the significance of the PD-L1 expression in esophageal squamous cell carcinoma (ESCC) patients receiving neoadjuvant chemoradiotherapy remains unclear. Methods: By means of PD-L1 immunohistochemistry 22C3 pharmDx assay, we evaluate the PD-L1 expression and its association with clinical outcome in 107 ESCC patients receiving neoadjuvant chemoradiotherapy. Results: Patients with positive PD-L1 expression have significantly lower pathological complete response rates (13% versus 32%; *p* = 0.036) than those with negative PD-L1 expression. Univariate survival analysis found that positive PD-L1 expression were correlated with poor overall survival (*p* = 0.004) and inferior disease-free survival (*p* < 0.001). In a multivariate analysis, positive PD-L1 expression was independently associated with the absence of a pathologically complete response (*p* = 0.044, hazard ratio: 3.542), worse overall survival (*p* = 0.006, hazard ratio: 2.017), and inferior disease-free survival (*p* < 0.001, hazard ratio: 2.516). Conclusions: For patients with ESCC receiving neoadjuvant chemoradiotherapy, positive PD-L1 expression independently predicts the poor chemoradiotherapy response and worse treatment outcome. Thus, our data suggests that PD-L1 may be an influential biomarker for prognostic classification and for immune checkpoint inhibitor therapies in ESCC patients receiving neoadjuvant chemoradiotherapy.

## 1. Introduction

In 2018, esophageal cancer was the sixth most common cause of cancer-related death worldwide [1]. In Asia, squamous cell carcinoma is the most prevalent histology of esophageal cancer. At diagnosis, patients with esophageal squamous cell carcinoma (ESCC) usually present with the advanced disease. The standard treatment modality for ESCC is esophagectomy. However, previous research has shown that the five-year overall survival rate of patients with advanced ESCC after receiving esophagectomy alone is only 20–30% [2,3,4,5]. Neoadjuvant chemoradiotherapy has been proposed for patients with advanced ESCC to reduce the primary tumor size and dispose of the micrometastases. Recent randomized trials and meta-analysis have revealed that neoadjuvant chemoradiotherapy followed by esophagectomy has a significant survival benefit compared to esophagectomy alone [6,7,8,9,10]. In particular, patients who achieved a pathological complete response following neoadjuvant chemoradiotherapy had improved survival odds than those who did not [11,12]. However, following neoadjuvant chemoradiotherapy, esophagectomy specimens show that only 20–40% patients can achieve pathological complete response [7,13,14], indicating that a large portion of patients do not respond to chemoradiotherapy. Furthermore, patients receiving neoadjuvant chemoradiotherapy followed by esophagectomy had higher morbidity and mortality rates postoperatively than those receiving esophagectomy alone [15]. Therefore, a biomarker is helpful to forecast the chemoradiotherapy response and its presence may shed light on novel target development.

The immune checkpoint proteins (programmed as cell death 1 (PD-1) receptor) and its ligand (programmed cell death ligand 1 (PD-L1)) are involved in the immune escape of cancer cells [16]. PD-L1 expressed on cancer cells binds to PD-1 receptor on T cells, which leads to T cell inactivation and exhaustion. This hampers cytokine production and causes T cell apoptosis. Cumulatively, these effects contribute to the growth of cancer cells [17,18]. Several studies have reported that PD-L1 overexpression predicts anticancer therapy resistances and poor treatment outcomes [19,20,21]. In addition, the use of PD-1/PD-L1 axis inhibitors in recent clinical trials had the meaningful activity and overall survival benefit in several types of cancers, including esophageal cancer [22,23,24,25]. Studies have also demonstrated that PD-L1 protein expression on the surface of cancer cells is associated with enhanced responses to PD-1/PD-L1 axis inhibitors [23,26]. However, the significance of PD-L1 expression in patients with ESCC after receiving neoadjuvant chemoradiotherapy followed by esophagectomy has not been investigated. Although biopsy specimens before treatment are often very small and show significant difference, they are the only tumor tissue samples for predicting clinical outcome in ESCC patients that have received neoadjuvant chemoradiotherapy. Therefore, we performed PD-L1 immunohistochemistry on pre-treatment biopsy specimens obtained from patients with advanced ESCC receiving neoadjuvant chemoradiotherapy followed by esophagectomy and then correlated the immunohistochemical results with treatment outcomes.

## 2. Materials and Methods

### 2.1. Patient and Tumor Materials

Between 1999 and 2013, ESCC patients that underwent neoadjuvant chemoradiotherapy followed by esophagectomy at Chang Gung Memorial Hospital and the Kaohsiung medical center were retrospectively analyzed. Patients without biopsy specimens before neoadjuvant chemoradiothrapy for immunohistochemistry were not allowed. The institutional review board of the Chang Gung Memorial Hospital approved the present study. The seventh American Joint Committee on Cancer (AJCC) staging system was used for clinical staging. The clinical staging was determined according to image examinations including a computed tomography (CT) scan of the abdomen and chest, endoscopic ultrasound (EUS), and/or positron emission tomography (PET) scan. The protocol of neoadjuvant chemoradiotherapy followed by esophagectomy was previously described [14,27]. Overall survival (OS) was computed from diagnosis date until the death date or the last follow-up. Disease-free survival (DFS) was calculated from the esophagectomy date until death due to any cause without recurrence evidence or date of recurrence. Ultimately, 107 ESCC patients who received neoadjuvant chemoradiotherapy followed by esophagectomy were enrolled for further analysis.

### 2.2. Immunohistochemistry (IHC)

The specimens were fixed in buffered formalin and embedded in paraffin. The IHC was carried out using standard reagents and techniques on a Dako Autostainer Link 48 platform (Agilent Technologies, Santa Clara, CA, USA) and EnVision FLEX visualization system (Agilent Technologies, citySanta Clara, CA, countryUSA). An automated IHC staining protocol of the PD-L1 IHC 22C3 pharmDx assay (Dako, Carpinteria, CA, USA) was verified with positive and negative controls used per manufacturer instructions. Briefly, in the PT Link (Dako PT100), deparaffinization, rehydration, and target antigen retrieval were performed through a three in one process. Then, specimens were incubated with the monocloncal mouse control IgG antibody (negative control) or anti-human PD-L1 monoclonal mouse antibody (clone 22C3), then with an anti-mouse linker antibody specific to the host species of the primary antibody, and then with a ready-to-use visualization reagent consisting of goat secondary antibody molecules and horseradish peroxidase molecules coupled with dextran. The enzymatic conversion of the subsequently added 3,3′-diaminobenzidine tetrahydrochloride (DAB) chromogen with color modification using a DAB enhancer resulted in precipitation of a visible reaction product at the site of antigen. The sections were then counterstained with hematoxylin and interpreted by a pathologist using a light microscope.

To determine the expression of PD-L1 protein, total viable tumor cells were evaluated and only tumors containing at least 100 viable tumor cells were scored. The positive staining was assessed in the context of non-specific background with 0 specific staining and <1+ intensity in the negative control reagent slide. The positivity of PD-L1 was represented as the complete circumferential or partial cell membrane staining of viable cancer cells. Tumor proportion score (TPS) was defined as the percentage of positive tumor cells over total tumor cells in the denominator. Tumor-associated immune cells or tumor cells with cytoplasmic staining were excluded from the scoring. Positive PD-L1 expression was defined as TPS > 1%. 

### 2.3. Statistical Analysis

Statistical analysis was performed using the SPSS 17 software package (manufactureIBM Corp., cityArmonk, NY, countryUSA). We used the chi-square test to compare the categorical data between the two groups. Logistic regression was used for the multivariate analysis of the pathological complete response. For univariate survival analysis in these patients, the Kaplan–Meier method was performed to plot the figures of OS and DFS. The difference between the two groups was evaluated by the log rank test. For multivariate survival analysis, the cox proportional hazards regression model was used. For every analysis, two-sided tests of significance were performed and the *p* value < 0.05 was considered as significant. 

## 3. Results

### 3.1. Patient Clinicopathological Characteristics

Table 1 shows the clinicopathological characteristics of these 107 patients. The median age of was 52 years (range: 37–77 years). The stage was revealed to be AJCC seventh stage II in 21 (20%) patients and AJCC seventh stage III in 86 (80%) patients. Meanwhile, the T classifications were T2 in 11 (10%) patients, T3 in 46 (43%) patients, and T4 in 50 (47%) patients. Additionally, 22 (21%) patients had N0 status, 36 (34%) patients had N1 status, 35 (33%) patients had N2 status, and 14 (13%) patients had N3 status. The locations of the primary tumor were as follows: upper esophagus in 20 (19%) patients, the middle esophagus in 43 (40%) patients, and the lower esophagus in 44 (41%) patients. In terms of a histologic grade, 22 (21%) patients were diagnosed with a grade 1 lesion, 58 (54%) patients were diagnosed with a grade 2 lesion, and 27 (25%) patients were diagnosed with a grade 3 lesion. Among these 107 patients receiving neoadjuvant chemoradiotherapy followed by esophagectomy, 28 (26%) patients achieved pathological complete response. The three-year OS and DFS rates of these 107 patients were 37% and 32%, respectively. 

**Figure 1:**
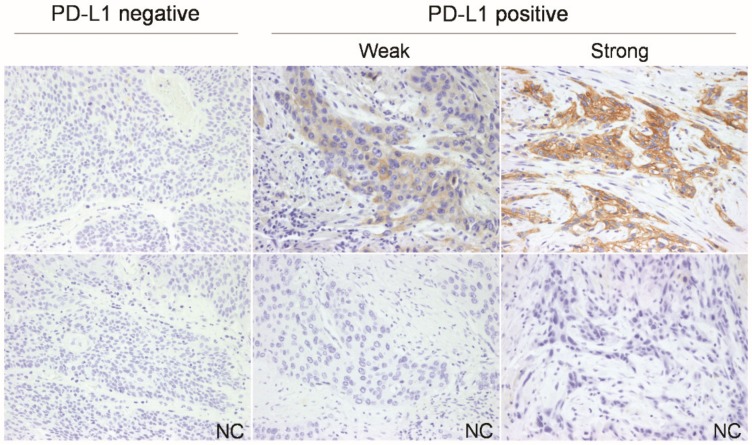
Representative photographs of PD-L1 immunostaining in esophageal squamous cell carcinoma. The positive staining was assessed against the negative control staining (NC) (original magnification 200x). PD-L1, programmed death-ligand 1.

As shown in Table 1, 75 (70%) and 32 (30%) patients were negative and positive for PD-L1 expression, respectively (Figure 1). There were no significant correlations between PD-L1 expression with histologic grade, primary tumor location, age, clinical T classification, clinical N classification, and clinical AJCC seventh staging (Table 2) 

### 3.2. Associations between Pathological Complete Respoznse with Clinicopathological Characteristics

Table 3 revealed the correlation between pathological complete responses with clinicopathological characteristics. We observed that positive PD-L1 expression (*p* = 0.036) and clinical T classification, T4 (*p* = 0.025) were significantly associated with the absence of pathological complete response. The multivariate analysis demonstrated that positive PD-L1 expression (*p* = 0.044, hazard ratio: 3.542, 95% confidence interval: 1.033–12.152) and clinical T classification, T4 (*p* = 0.047, hazard ratio: 3.225, 95% confidence interval: 1.016–10.231), were independently associated with the absence of a pathologically complete response.

### 3.3. Associations between Patient Survival with Clinicopathological Characteristics 

**Figure 2:**
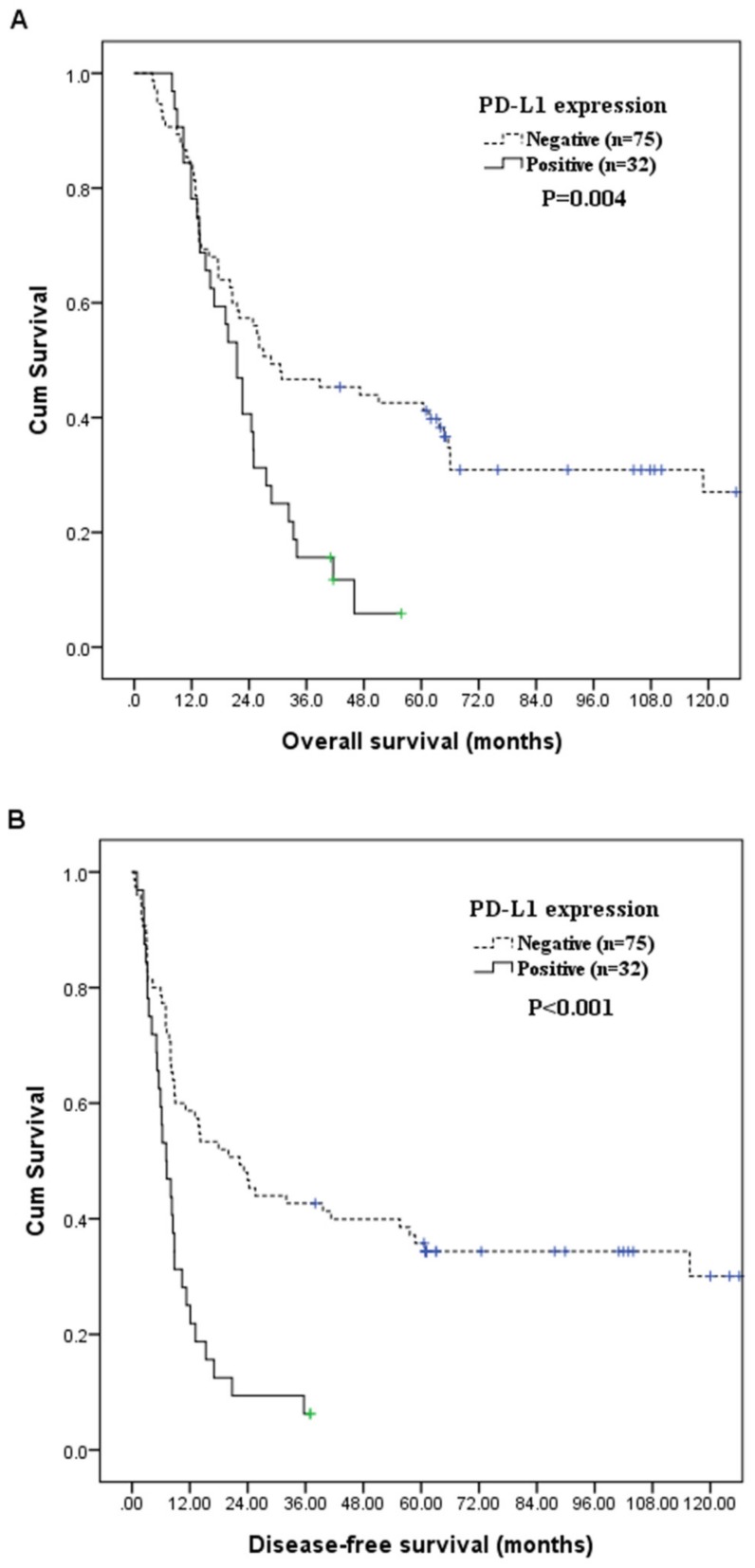
(**A**) Overall survival according to PD-L1 expression. (**B**) Disease-free survival according to PD-L1 expression. PD-L1, programmed death-ligand 1.

Table 4 shows the association between OS and DFS with clinicopathological characteristics and PD-L1 expression. We found that positive PD-L1 expression (*p* = 0.004; Figure 2A), clinical T classification, T4 (*p* = 0.015), and clinical N classification, N2/3 (*p* = 0.025) were correlated with inferior OS significantly at univariate level. Besides, univariate analysis also revealed that positive PD-L1 expression (*p* < 0.001; Figure 2B), clinical T classification, T4 (*p* = 0.006), and clinical N classification, N2/3 (*p* = 0.044) were significantly correlated with poor DFS. In a multivariate analysis, positive PD-L1 expression (*p* = 0.006, hazard ratio: 2.017, 95% confidence interval: 1.223–3.326) and clinical T classification, T4 (*p* = 0.022, hazard ratio: 1.910, 95% confidence interval: 1.097–3.325), were independently poor prognosticators for inferior OS. Meanwhile, positive PD-L1 expression (*p* < 0.001, hazard ratio: 2.516, 95% confidence interval: 1.537–4.120) and clinical T classification, T4 (*p* = 0.03, hazard ratio: 1.857, 95% confidence interval: 1.060–3.252), were independently associated with worse DFS. The three-year OS rates were 47% in patients with negative PD-L1 expression, and 16% in patients with positive PD-L1 expression. The three-year DFS rates were 43% in patients with negative PD-L1 expression, and 6% in patients with positive PD-L1 expression.

## 4. Discussion

Previous studies [26,28] have described that positive PD-L1 expression is correlated with worse prognosis in numerous human cancers and also suggested that such expression could serve as a biomarker which predicts the response to anti-PD-1/PD-L1 therapies. However, the significance of PD-L1 expression in patients with ESCC receiving neoadjuvant chemoradiotherapy followed by esophagectomy remain largely undefined. Therefore, we conducted the current study in order to determine the significance of PD-L1 expression in ESCC patients who underwent neoadjuvant chemoradiotherapy followed by esophagectomy.

In the present study, positive PD-L1 expression was noted in 32 (30%) of 107 patients with ESCC. Hatogai et al. [29] reported that positive PD-L1 expression was noted in 67 (23.4%) of 286 patients with ESCC receiving curative surgical resection. Meanwhile, a recent meta-analysis [30] showed that positive PD-L1 expression was observed in 559 (41.4%) of 1350 patients with ESCC. The discrepancy between these studies may have resulted from differences in the PD-L1 antibodies and IHC assessment methods used. 

In our study, we did not observe a significant correlation between histologic grades with pathologically complete responses, despite a previous study by Tamaoki et al. [31] that reported a single-minded 2 (SIM2) increased chemoradiotherapy sensitivity through tumor differentiation in ESCC. However, we found that positive PD-L1 expression was significantly associated with the absence of pathological complete response. We observed that pathologically complete responses after neoadjuvant chemoradiotherapy was noted in 24 (32%) of the 75 patients with negative PD-L1 expression. However, 4 (13%) of the 32 patients with positive PD-L1 expressions achieved a pathologically complete response. For patients with locally advanced ESCC, multimodality treatment including definitive chemoradiotherapy or neoadjuvant chemoradiotherapy followed by esophagectomy has been commonly used. The ability to distinguish responders from non-responders could provide more suitable multimodality treatment options.

Our study revealed that a positive PD-L1 expression is an independent prognosticator in patients with advanced ESCC treated with preoperative chemoradiotherapy. Kudo et al. [25] showed that the use of the anti-PD-1 antibody, nivolumab, in 65 chemotherapy-refractory ESCC patients showed promising activity, with a 17% objective response rate and a manageable safety profile. Tanaka et al. [32] first reported that many interferon-gamma-inducible genes including PD-L1 and cytotoxic T-lymphocyte markers such as perforin (PRF1) and granzyme B (GZMB) were activated by chemoradiotherapy in ESCC. Lim et al. [33] acquired 19 paired ESCC tumor tissues before and after preoperative chemoradiotherapy and found that PD-L1 expression in ESCC cells increased after preoperative chemoradiotherapy. The recent phase III PACIFIC study [34] reported that after chemoradiotherapy, the administration of the anti-PD-L1 antibody, durvalumab, can ameliorate progression-free survival in non-small-cell lung cancer patients with unresectable stage III disease. Taken together, these previous studies and our findings highlight the potential for a combination of anti-PD-1/PD-L1 therapy and chemoradiotherapy in patients with advanced ESCC.

The present study has limitations. The patient number in our study is relatively small. Our analysis was retrospective. Furthermore, we did not evaluate PD-L1 expression in immune cells because some of the biopsy specimens were too small to have enough cells evaluated. Besides, biopsy specimens are too small to show significant difference. For clinical use, the cut-off value should be determined by extensive IHC using multiple sections in the multi-institutional cohorts in the future. 

## 5. Conclusions

In conclusion, our study showed that positive PD-L1 expression independently predicts poor response to chemoradiotherapy and worse survival of patients with ESCC receiving neoadjuvant chemoradiotherapy followed by esophagectomy. Therefore, PD-L1 might be a potential target in advanced ESCC patients receiving chemoradiotherapy.

## Figures and Tables

**Table 1:** Clinicopathologic features of 107 patients with locally advanced esophageal squamous cell carcinoma who received preoperative chemoradiotherapy.

Parameters	No. of Cases (Percentage)
Age (years) (mean: 53.6, median: 52, range 37–77)	
<50	40 (37%)
50 ≤ Age < 60	36 (34%)
60 ≤ Age< 70	27 (25%)
70 ≤ Age	4 (4%)
Clinical seventh American Joint Committee on Cancer (AJCC) stage	
II	21 (20%)
III	86 (80%)
Clinical T classification	
T2	11 (10%)
T3	46 (43%)
T4	50 (47%)
Clinical N classification	
N0	22 (21%)
N1	36 (34%)
N2	35 (33%)
N3	14 (13%)
Histologic grade (Tumor differentiation)	
Grade 1 (Well differentiated)	22 (21%)
Grade 2 (Moderately differentiated)	58 (54%)
Grade 3 (poorly differentiated, undifferentiated)	27 (25%)
Primary tumor location	
Upper	20 (19%)
Middle	43 (40%)
Lower	44 (41%)
PD-L1 expression	
Negative	75 (70%)
Positive	32 (30%)
pCR	
Absent	79 (74%)
Present	28 (26%)

PD-L1, programmed death-ligand 1; pCR, pathological complete response.

**Table 2:** Associations between PD-L1 expression and clinicopathologic parameters.

Parameters		PD-L1 Expression		
		Negative	Positive	*p* value
Age	≤52 years old	40	15	0.54
	>52 years old	35	17	
Clinical seventh AJCC stage	II	15	6	0.88
	III	60	26	
Clinical T classification	T2/3	39	18	0.69
	T4	36	14	
Clinical N classification	N0	18	4	0.18
	N1/2/3	57	28	
Clinical N classification	N0/1	40	18	0.78
	N2/3	35	14	
Histologic grade	Grade 1/2	56	24	0.97
	Grade 3	19	8	
Histologic grade	Grade 1	18	4	0.18
	Grade 2/3	57	28	
Primary tumor location	Upper/Middle	44	19	0.95
	Lower	31	13	

PD-L1, programmed death-ligand 1. x^2^ test was used for statistical analysis.

**Table 3:** Associations between a pathologically complete response and clinicopathologic parameters.

**Parameters**		**Pathological Complete Response**		
		Present	Absent	*p* value
Age	≤52 years old	11	44	0.14
	>52 years old	17	35	
Clinical seventh AJCC stage	II	8	13	0.17
	III	20	66	
Clinical T classification	T2/3	20	37	0.025*
	T4	8	42	
Clinical N classification	N0	9	13	0.078
	N1/2/3	19	66	
Clinical N classification	N0/1	17	41	0.42
	N2/3	11	38	
Histologic grade	Grade 1/2	19	61	0.87
	Grade 3	6	21	
Histologic grade	Grade 1	8	14	0.22
	Grade 2/3	20	65	
Primary tumor location	Upper/Middle	12	51	0.21
	Lower	13	31	
PD-L1 expression	Negative	24	51	0.036*
	Positive	4	28	

PD-L1, programmed death-ligand 1. * Statistically significant. x^2^ test or Fisher’s exact test was used for statistical analysis.

**Table 4:** Results of univariate log-rank analysis of prognostic factors for overall survival and disease-free survival in 107 patients with locally advanced esophageal squamous cell carcinoma who received preoperative chemoradiotherapy.

Factors	No. of Patients	Overall Survival (OS)		Disease-Free Survival (DFS)	
		3-Year OS Rate (%)	*p* Value	3-Year DFS Rate (%)	*p* Value
Age					
≤52 years old	55	35%	0.51	29%	0.86
>52 years old	52	40%		35%	
Clinical seventh AJCC stage					
II	21	52%	0.14	52%	0.084
III	86	34%		27%	
Clinical T classification					
T2/3	57	49%	0.015*	42%	0.006*
T4	50	24%		20%	
Clinical N classification					
N0	22	55%	0.10	55%	0.046*
N1/2/3	85	33%		26%	
Clinical N classification					
N0/1	58	47%	0.025*	38%	0.044*
N2/3	49	27%		25%	
Histologic grade					
Grade 1/2	80	39%	0.18	33%	0.82
Grade 3	27	33%		30%	
Histologic grade					
Grade 1	22	50%	0.17	50%	0.24
Grade 2/3	85	34%		27%	
Primary tumor location					
Upper/Middle	63	38%	0.89	30%	0.75
Lower	44	36%		34%	
PD-L1 expression					
Negative	75	47%	0.004*	43%	<0.001*
Positive	32	16%		6%	

PD-L1, programmed death-ligand 1. *Statistically significant.
